# High-power dual-wavelength Ho-doped fiber laser at >2 μm tandem pumped by a 1.15 μm fiber laser

**DOI:** 10.1038/srep42402

**Published:** 2017-02-09

**Authors:** Xiaoxi Jin, Zhaokai Lou, Yizhu Chen, Pu Zhou, Hanwei Zhang, Hu Xiao, Zejin Liu

**Affiliations:** 1 College of Optoelectronic Science and Engineering, National University of Defense Technology, Changsha 410073, China; 2 Hunan Provincial Collaborative Innovation Center of High Power Fiber Laser, Changsha 410073, China

## Abstract

We demonstrated a high-power continuous-wave (CW) dual-wavelength Ho-doped fiber laser (HDFL) at 2049 nm and 2153 nm with a simple coupled-cavity configuration. A ~100 W laser diode-pumped fiber laser at 1150 nm served as the pump source. The maximum output power reached ~22.3 W and the slope efficiency was 23%. By altering the incident pump power, the power ratio of two signal wavelengths could be tuned in a large range due to gain competition. As far as we know, this is the first CW dual-wavelength HDFL with the power exceeding ten-watt-level, and the first dual-wavelength HDFL with the central wavelengths exceeding 2.0 μm and 2.15 μm respectively.

2 μm fiber lasers have attracted a large amount of attentions in recent decades, attributed to applications in LIDAR, gas sensing, medicine, material processing and nonlinear optics^1^,^2^,^3^,^4^,^5^,^6^. With the rapid development of 2 μm fiber-based devices and high-power pump sources, the power records of Tm- and Ho-doped silica fiber lasers operating at 2 μm have reached hundred- to kilo-watt level^7^,^8^,^9^. Besides these remarkable milestones, fiber laser techniques to achieve dual-wavelength lasing at 2 μm are also under considerable research, due to the prospective applications in the fields including next-generation optical fiber communication networks, ranging systems, spectroscopy, terahertz generation and mitigating stimulated Brillouin scattering effects in fiber amplifiers^10^,^11^,^12^,^13^,^14^,^15^,^16^,^17^.

Dual-wavelength lasing at 2 μm could be realized by utilizing Tm- or Ho-doped silica fiber. Rapid progress of 2 μm dual-wavelength fiber lasers has been made in recent years. Different approaches have been employed in continuous-wave (CW) Tm- or Tm-Ho-co-doped fiber lasers to obtain stable dual-wavelength lasing, including techniques based on volume Bragg gratings^10^, coupled laser cavities with cascaded fiber Bragg gratings (FBGs)^11^,^12^, polarization hole burning by the means of high-birefringence fiber Bragg gratings (HB-FBGs)^13^ or birefringence gain medium^14^, cascaded filter^15^, parallel connection of FBGs by using coupler^16^, using photonic crystal fiber (PCF)^17^ and multi-mode non-adiabatic taper fiber as wavelength-selective filter. While for pulsed fiber lasers at 2 μm, carbon nanotube (CNT) saturable absorber^18^ and nonlinear optical loop mirror (NOLM)^19^ have been employed in passive mode-locked fiber lasers to generate dual wavelengths.

High-power dual-wavelength fiber lasers with the operating wavelength at >2 μm have potentials to be employed in the field of optical communications, remote sensing, or as a novel pump laser for dual-wavelength mid-infrared optical parametric oscillator. However, in these reported techniques to achieve dual-wavelength operation around 2 μm, the output powers were relatively low (less than ~100 mW) and the wavelengths were generally confined below 2.0 μm, which bring obstacles for the potential applications preferring powerful dual-wavelength laser sources with excellent property of propagation in the atmosphere. Only a few approaches succeeded in generating dual-wavelength laser exceeding 2.0 μm^15^,^18^. In 2012, A. Chamorovskiy *et al*. demonstrated a mode-locked Ho-doped fiber laser (HDFL) based on CNT saturable absorber, which could emit dual-wavelength soliton pulse at 2050 nm and 2075 nm with average power of 40 mW^18^. In 2016, S. Fu *et al*. reported a stable dual-wavelength Tm-Ho-co-doped fiber laser based on cascaded single-mode-multi-mode-single-mode (SM-MM-SM) structures^15^, in which dual-wavelength fiber laser at 2002.8 nm and 2016.1 nm with 4.9 mW output power was achieved. Therefore, to further extend the applications fields of 2 μm dual-wavelength fiber lasers, high power and long operating wavelengths are in great demand.

Ho-doped silica fiber is a favorable gain medium to generate fiber laser above 2.0 μm, with longer operating wavelengths (~2.05–2.15 μm) than Tm-doped silica fiber (~1.90–2.05 μm). Powerful fiber lasers at 1.15 μm or 1.9 μm can be used as pump sources of HDFLs, which enables high power emission^9^,^20^,^21^,^22^,^23^,^24^. According to previous achievements^12^, coupled cavity formed by large-mode-area (LMA) active fiber and two pairs of FBGs was a feasible approach to obtain high-power dual-wavelength operation at selected wavelengths. However, to the best of our knowledge, it has not been reported yet that achieving high-power dual-wavelength fiber lasers at >2 μm by employing coupled cavity and Ho-doped silica fiber.

We reported a 100 W-level fiber at 1150 nm previously by simultaneously employing Yb and Raman gain^25^, which is a suitable pump source of HDFL. In this paper, we demonstrated the first high-power CW dual-wavelength HDFL in an all-fiber coupled-cavity configuration using this powerful home-made fiber laser at 1150 nm as pump laser. The maximum output power reached ~22.3 W and the slope efficiency was 23% with center wavelengths at 2049 nm and 2153 nm. We demonstrated the power transfer process of dual-wavelength HDFL with large spectral spacing of exceeding 100 nm during the power scaling due to gain competition. The tunable range of power ratio was also discussed.

## Laser Configuration

**Figure 1:**
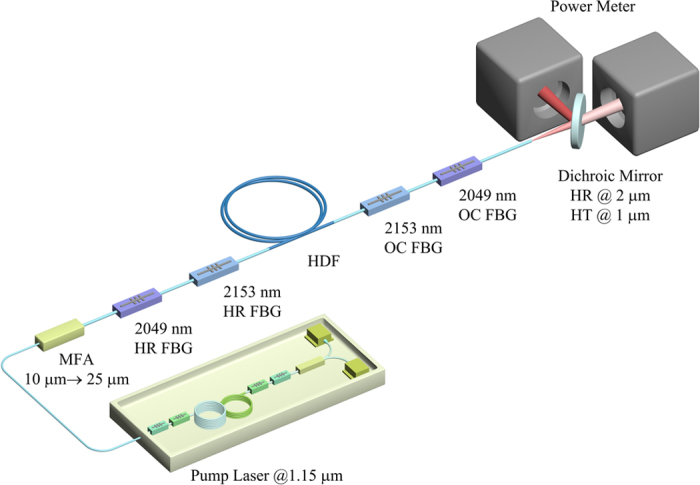
Experimental setup of dual-wavelength Ho-doped fiber laser pumped by a high-power 1150 nm fiber laser. MFA: mode field adapter; HR: high-reflectivity; OC: output-coupler; FBG: fiber Bragg grating; HDF: Ho-doped fiber; HT: high-transmittance.

The experimental setup of the dual-wavelength HDFL is schematically depicted in Fig. 1. This HDFL consists of a powerful 1150 nm pump laser, a mode field adapter (MFA) to match different fiber geometries, a coupled cavity formed by two pairs of FBGs, and a piece of Ho-doped fiber. All the fiber-based devices were fusion spliced to ensure the HDFL compact and robust. The pump laser emitting from the 1150 nm fiber laser was directly launched into the core via a MFA. The fiber geometry of input port and output port of MFA were 10/125 μm and 25/250 μm respectively (diameter of core/inner cladding), which was used to match the pigtail of 1150 nm pump laser and the input port of HDFL. The pigtail of pump laser was 10/125 μm double-cladding fiber. The fiber geometries of Ho-doped fiber and pigtails of FBGs were all 25/250 μm. The coupled cavity of this HDFL was formed by two pairs of FBGs. One pair centered at 2049 nm with reflectivity of 99% and 10% respectively, and the other centered at 2153 nm with the reflectivity of 99% and 59%. The 3 dB bandwidths of 2049 nm FBGs were 2.0 nm and 1.6 nm, respectively. And the 3 dB bandwidths of 2153 nm FBGs were 1.3 nm and 1.0 nm. A piece of 1.0 m double-cladding HDF served as the gain medium of dual-wavelength HDFL, which was in the center of coupled cavity (see Fig. 1). The core absorption coefficient of HDF was ~20 dB/m at 1150 nm. The fusion splices between Ho-doped fiber, 2153 nm OC (output coupler) FBG and 2049 nm OC FBG were coated by high-refractive-index gel to strip unabsorbed pump light. All the fusion splices and fiber-based devices of HDFL were fixed on a water-cooled heat sink to dissipate heat load in fiber. The output pigtail of 2049 nm OC FBG was angle-cleaved to avoid unwanted feedback from fiber facet.

To separate signal laser at 2 μm and residual pump laser at 1.15 μm emitting from the angle-cleaved output port of HDFL, a dichroic mirror with high reflectivity at ~2 μm and high transmittance at ~1 μm was used. All the spectra and powers shown below were measured and recorded from the reflected signal laser. We used a piece of multi-mode fiber to couple the signal laser into an optical spectrum analyzer (OSA) with 0.2 nm resolution to analyze output spectra of the dual-wavelength HDFL. Power meters were employed to measure the power of signal laser reflected by the dichroic mirror.

## Results and Discussion

**Figure 2:**
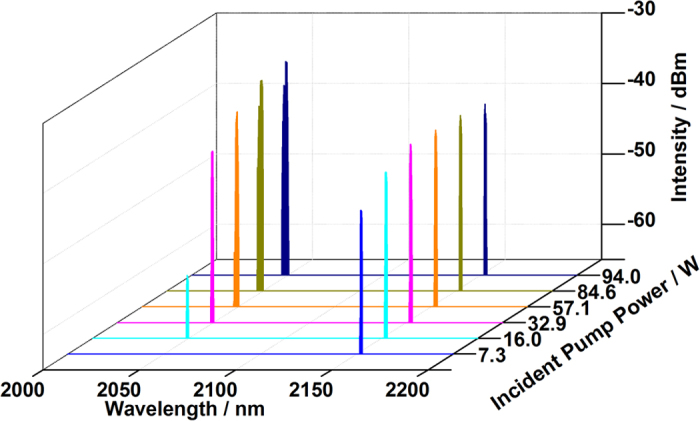
Optical spectra of coupled-cavity HDFL with various pump powers.

Due to the gain competition in the dual-wavelength coupled-cavity HDFL, the powers and spectra of output lasers varied with the increase of pump laser, especially when the spectral spacing was as high as 104 nm which indicates large difference of cross sections. The optical spectra with various incident 1150 nm pump powers are shown in Fig. 2. Two peaks at 2049 nm and 2153 nm were observed simultaneously in the experiment, which corresponded to the reflectivity spectra of FBGs. As the reflectivity of 2153 nm OC FBG (59%) is much higher than that of 2049 nm OC FBG, it is easy to meet the requirements of resonance at 2153 nm in this coupled cavity despite the low emission cross section. Thus, when the pump power was lower than 16 W, only 2153 nm laser emitted. Then 2049 nm laser emerged when more pump power was launched into cavity. With the further increase of pump power, both gains at 2049 nm and 2153 nm became stronger which lead to gain competition among limited excited Ho ions. Amplified spontaneous emission (ASE) and parasitic oscillation were suppressed efficiently, even though 2153 nm is near the long-wavelength gain spectrum edge of silica-based HDF. To clarify the process of power transfer and gain competition during the power scaling of dual-wavelength HDFL, we calculated and analyzed the ratios and powers of lasers at 2049 nm and 2153 nm under different pump powers individually.

**Figure 3:**
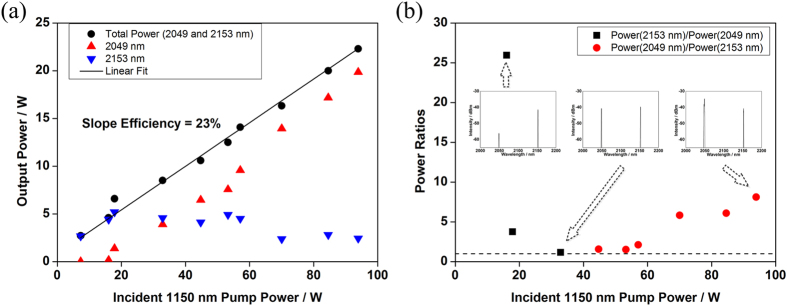
Powers and ratios of dual-wavelength HDFL under different pump powers. (**a**) Powers at 2049 nm (red upper triangles) and 2153 nm (blue lower triangles) of dual-wavelength HDFL, and total output power (black circles). (**b**) Power ratios of dual-wavelength HDFL. Insets in (**b**) are spectra with various pump powers of 16 W, 32 W and 94 W, respectively.

By numerical integrating the spectra in Fig. 2, we obtained power ratios of lasers at 2049 nm and 2153 nm to total powers. Therefore, powers at 2049 nm and 2153 nm could be estimated after measuring the total output power of dual-wavelength HDFL. The output powers and ratios of dual-wavelength HDFL under different pump powers are shown in Fig. 3. As shown in Fig. 3(a), the maximum output power of dual-wavelength HDFL was ~22.3 W with ~20 W at 2049 nm and ~2.3 W at 2153 nm, in which lasers at 2049 nm took a dominant place. The slope efficiency of this dual-wavelength HDFL was 23% with respect to incident 1150 nm pump power, which is comparable to the efficiency of reported HDFLs operating at common wavelengths (~2.05–2.10 μm) when core-pumped by diode^26^, Yb-doped fiber laser^20^, Raman fiber laser^21^ or random distributed feedback fiber laser^22^ at 1.15 μm. The power of 2153 nm laser peaked at ~5 W and lasing at 2049 nm was just observed, when the incident pump power reached ~18 W. By further increasing the pump power, laser power at 2049 nm continuously improved while laser power at 2153 nm was in a downward trend. When the pump power was 32 W, the power of 2049 nm and 2153 nm was equally at ~4 W. The power ratios of dual-wavelength HDFL was shown in Fig. 3(b). The dashed line was added in the figure to denote unity power ratio, which means that the signal powers at 2049 nm and 2153 nm are equal. When the pump power was around the threshold of 2049 nm laser, power ratio of 2153 nm to 2049 nm was measured as high as 26 times. The corresponding spectrum was shown in the first inset of Fig. 3(b). When we increased the pump power to 32 W, the dual-wavelength lasing with equal optical intensity was achieved, and the spectrum was shown in the second inset. Due to the increased gain at 2049 nm with improved pump power, power of 2049 nm exceeded that of 2153 nm when the pump power beyond 32 W. At the maximum pump power of 94 W, the power of 2049 nm laser was improved to 8 times of 2153 nm laser, and the spectrum was demonstrated in the last inset. Thus, in the current dual-wavelength HDFL with the HDF length of 1.0 m and corresponding reflectivity of coupled cavity, the power ratio of two signal wavelengths could be easily tuned by altering the pump power.

**Figure 4:**
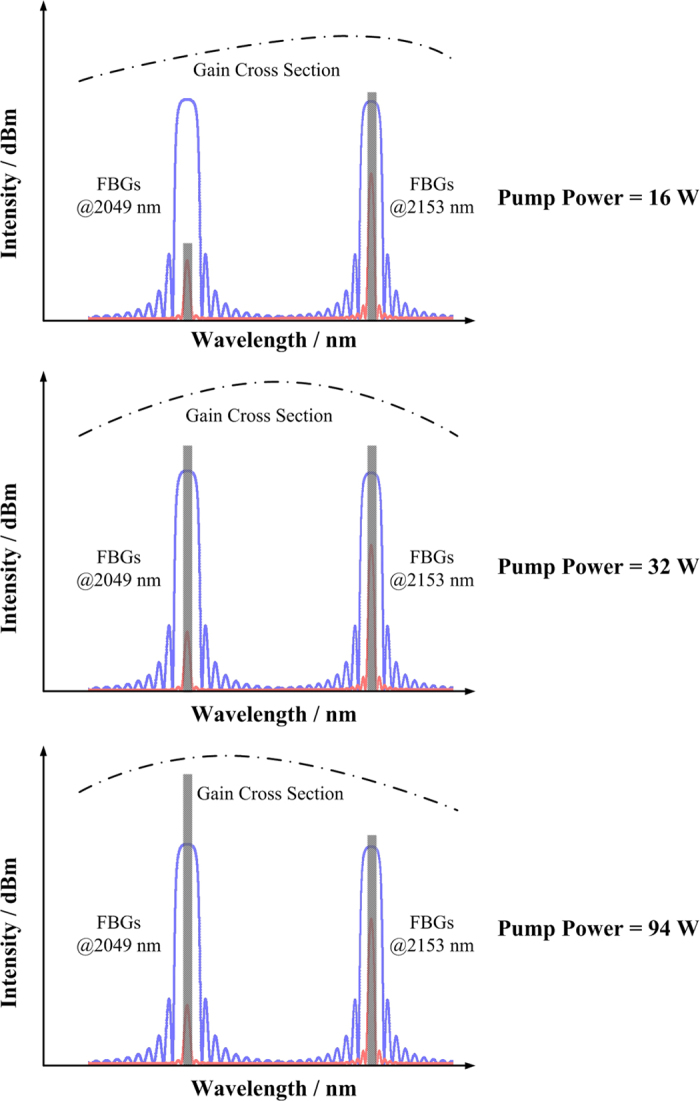
Schematic diagram of the dual-wavelength HDFL in a coupled-cavity configuration under different pump powers. The gain cross sections of Ho ions in silica fiber are schematically shown in dash dot lines. The reflectivities of HR and OC FBGs were shown in blue and red lines, respectively.

The gain competition between lasers at 2049 nm and 2153 nm could be illustrated schematically in Fig. 4. As we know, the gain cross section changed with population of excited ions, thus different pump powers result in different gain cross sections. With the increase of excited Ho ions population, the gain cross section at 2049 nm will eventually surpass that at 2153 nm in Ho:silica^27^. Moreover, in the dual-wavelength HDFL, the reflectivity of OC FBG at 2153 nm was 59%, which is much higher than that of OC FBG at 2049 nm (10%). Therefore, when the incident pump power was low (as the case of 16 W pump power), the net gain of 2153 nm laser was higher, and 2153 nm laser emitted with several watt power while 2049 nm laser was just above the threshold. As the case of 32 W pump power in the experiment, the gain cross section at 2049 nm increased more rapidly and surpassed that of 2153 nm laser, which balanced the cavity loss and led to equal net gain at two wavelengths in the coupled cavity. Once the pump power increases beyond that ‘balanced point’, laser at 2049 nm will take the dominant place afterwards as the case of 94 W pump power. As a result, it is feasible to further increase the equal power level of two wavelengths by increasing that ‘balanced point’, such as enhancing the reflectivity of 2153 nm OC FBG, and/or decreasing the reflectivity of 2049 nm OC FBG. In the current dual-wavelength HDFL, the maximum output power and power-ratio-tunable range are limited by parameters including active fiber length and FBG reflectivity. Besides, the coupled-cavity configuration is flexible to achieve CW dual-wavelength or multi-wavelength lasing by replacing the FBG pairs with different central wavelengths, and the principle on choosing cavity parameters are similar as mentioned above.

**Figure 5:**
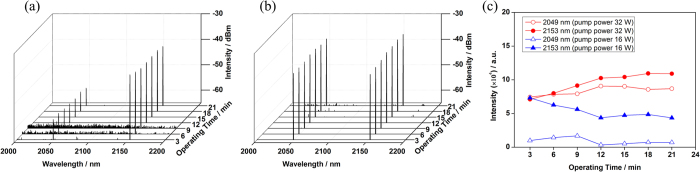
Dual-wavelength operation of coupled-cavity HDFL under two different pump powers. (**a**): 16 W pump power; (**b**): 32 W pump power; (**c**) Fluctuations of coupled optical intensity.

We also measured the spectral stability of this dual-wavelength HDFL and the results are depicted in Fig. 5. When monitoring the spectra, a fixed multi-mode fiber was employed to couple part of output signal laser into OSA. As stated previously, the HDFL just started to operate in dual-wavelength mode when the pump power reached 16 W, and the powers at two signal wavelengths became nearly equal when 32 W pump power launched into the coupled cavity. The optical spectra under the pump power of 16 W and 32 W with more than 20 minutes operating time are shown in Fig. 5 (a) and (b), respectively. There weren’t any ASE or parasitic oscillation observed in the range of 2000 nm to 2200 nm, which indicated the spectral purity of dual-wavelength HDFL. In Fig. 5(b), the optical signal-to-noise ratios (SNR) for both wavelengths were kept exceeding 25 dB. By integrating the spectra in Fig. 5 (a) and (b), the fluctuations of coupled dual-wavelength optical intensity are shown in Fig. 5(c). Mode competition is believed as the main reason that leads to power fluctuation in the dual-wavelength fiber laser, which could get weaken when the gain saturation occurred such as by improving pump power^28^. In this dual-wavelength HDFL, the power fluctuation decreased to 1.8 dB after improving the pump power to 32 W.

## Conclusion

In conclusion, we reported the first high-power CW dual-wavelength HDFL at 2 micron with the power exceeding ten-watt level, and the first dual-wavelength HDFL with the center wavelengths exceeding 2.0 μm and 2.15 μm, respectively. A 100 W-level laser diode-pumped fiber laser at 1150 nm served as the pump laser. The dual-wavelength HDFL peaked at 2049 nm and 2153 nm. The maximum output power reached ~22.3 W and the slope efficiency was 23% with respect to incident pump power. The dual-wavelength power ratio could be tuned in a large range by changing the pump power in the coupled-cavity configuration with the help of gain competition. Moreover, such high-power and power-ratio-tunable dual-wavelength HDFL is favorable to be employed as seed laser of dual-wavelength MOPA to realize high power emission with large spectral spacing, or be employed as seed laser of nonlinear MOPA^29^,^30^ to achieve powerful lasing at long wavelength with small gain cross section, which has many prospective applications such as optical communication at 2 microns. Also, the high-power dual-wavelength HDFL could also be used as a compact pump source of optical parametric oscillator.

## Additional Information

**How to cite this article**: Jin, X. *et al*. High-power dual-wavelength Ho-doped fiber laser at >2 µm tandem pumped by a 1.15 µm fiber laser. *Sci. Rep.* **7**, 42402; doi: 10.1038/srep42402 (2017).

**Publisher's note:** Springer Nature remains neutral with regard to jurisdictional claims in published maps and institutional affiliations.

## References

[b1] Jackson S. D. Towards High-Power Mid-Infrared Emission From a Fibre Laser. Nat Photonics 6, 423–431 (2012).

[b2] Hemming A., Simakov N., Haub J. & Carter A. A Review of Recent Progress in Holmium-Doped Silica Fibre Sources. Opt Fiber Technol 20, 621–630 (2014).

[b3] Geng J., Wang Q., Lee Y. & Jiang S. Development of Eye-Safe Fiber Lasers Near 2 μm. IEEE J Sel Top Quant 20, 150–160 (2014).

[b4] Yan Z. . Widely Tunable Tm-doped Mode-Locked All-Fiber Laser. Sci Rep-UK 6, 27245 (2016). 10.1038/srep27245 PMC4893705 27263655

[b5] Wen X. . Highly Tm3+ Doped Germanate Glass and its Single Mode Fiber for 2.0 μm Laser. Sci Rep-UK 6, 20344 (2016). 10.1038/srep20344 PMC4734336 26828920

[b6] Tang Y. & Xu J. A Random Q-switched Fiber Laser. Sci Rep-UK 5, 9338 (2015). 10.1038/srep09338 PMC4369747 25797520

[b7] Goodno G. D., Book L. D. & Rothenberg J. E. Low-Phase-Noise, Single-Frequency, Single-Mode 608 W Thulium Fiber Amplifier. Opt Lett 34, 1204–1206 (2009). 19370118 10.1364/ol.34.001204

[b8] Ehrenreich T. . 1-kW, all-glass Tm: fiber laser. Fiber Lasers VII: Technology, Systems, and Applications, San Francisco, California, USA. Proceedings of SPIE (International Society for Optics and Photonics). (2010, January 23).

[b9] Hemming A. . A Monolithic Cladding Pumped Holmium-Doped Fibre Laser. CLEO: Science and Innovations. San Jose, California, USA. OSA Technical Digest (Optical Society of America). (doi: 10.1364/CLEO_SI.2013.CW1M.1) (2013, June 9-14).

[b10] Wang F., Shen D., Fan D. & Lu Q. Widely Tunable Dual-Wavelength Operation of a High-Power Tm: Fiber Laser Using Volume Bragg Gratings. Opt Lett 35, 2388–2390 (2010). 20634839 10.1364/OL.35.002388

[b11] Zhou P., Wang X. L., Ma Y. X., Han K. & Liu Z. J. Stable All-Fiber Dual-Wavelength Thulium-Doped Fiber Laser and its Coherent Beam Combination. Laser Phys 21, 184–187 (2011).

[b12] Wang Y., Zhou Y., Yan S., Tang Y. & Xu J. Dual-Wavelength 2-μm Fiber Laser with Coupled Fiber Bragg Grating Cavities. IEEE Photonic Tech L 28, 1193–1196 (2016).

[b13] Liu S. . Tunable Dual-Wavelength Thulium-Doped Fiber Laser by Employing a HB-FBG. IEEE Photonic Tech L 26, 1809–1812 (2014).

[b14] Ma X., Luo S. & Chen D. Switchable and Tunable Thulium-Doped Fiber Laser Incorporating a Sagnac Loop Mirror. Appl Optics 53, 4382–4385 (2014). 10.1364/AO.53.004382 25090056

[b15] Fu S. . Dual-Wavelength Fiber Laser Operating Above 2 μm Based On Cascaded Single-Mode-Multimode-Single-Mode Fiber Structures. Opt Express 24, 11282–11289 (2016). 27410059 10.1364/OE.24.011282

[b16] Yang W., Lu P., Wang S., Liu D. & Zhang J. 2-μm Switchable, Tunable and Power-Controllable Dual-Wavelength Fiber Laser Based On Parallel Cavities Using 3 × 3 Coupler. Applied Physics B 120, 349–354 (2015).

[b17] Soltanian M. R. K. . A Stable Dual-wavelength Thulium-doped Fiber Laser at 1.9 μm Using Photonic Crystal Fiber. Sci Rep-UK 5, 14537 (2015). 10.1038/srep14537 PMC4600979 26455713

[b18] Chamorovskiy A. Y., Marakulin A. V., Kurkov A. S. & Okhotnikov O. G. Tunable Ho-doped Soliton Fiber Laser Mode-Locked by Carbon Nanotube Saturable Absorber. Laser Phys Lett 9, 602 (2012).

[b19] Yan Z. . Tunable and Switchable Dual-Wavelength Tm-doped Mode-Locked Fiber Laser by Nonlinear Polarization Evolution. Opt Express 23, 4369–4376 (2015). 25836473 10.1364/OE.23.004369

[b20] Kurkov A. S., Dvoyrin V. V. & Marakulin A. V. All-Fiber 10 W Holmium Lasers Pumped at λ = 1.15 μm. Opt Lett 35, 490–492 (2010). 20160794 10.1364/OL.35.000490

[b21] Wang X. . Raman Fiber Laser-Pumped High-Power, Efficient Ho-doped Fiber Laser. J Opt Soc Am B 31, 2476–2479 (2014).

[b22] Zhang H. . Hundred-Watt-Level High Power Random Distributed Feedback Raman Fiber Laser at 1150 nm and its Application in Mid-Infrared Laser Generation. Opt Express 23, 17138–17144 (2015). 26191722 10.1364/OE.23.017138

[b23] Wang X. . High Power, Compact, Passively Q-switched Ho-doped Fiber Laser Tandem Pumped by a 1150 nm Raman Fiber Laser. Laser Phys Lett 11, 95101 (2014).

[b24] Jin X., Wang X., Wang X., Xiao H. & Zhou P. High-Power Ho-doped All-Fiber Superfluorescent Source Pumped by a 1150 nm Raman Fiber Laser. Appl Optics 53, 8302–8304 (2014). 10.1364/AO.53.008302 25608073

[b25] Chen Y., Xiao H., Xu J., Leng J. & Zhou P. Laser Diode-Pumped Dual-Cavity High-Power Fiber Laser Emitting at 1150 nm Employing Hybrid Gain. Appl Optics 55, 3824–3828 (2016). 10.1364/AO.55.003824 27168299

[b26] Jackson S. D., Bugge F. & Erbert G. Directly Diode-Pumped Holmium Fiber Lasers. Opt Lett 32, 2496–2498 (2007). 17767283 10.1364/ol.32.002496

[b27] Simakov N., Hemming A., Clarkson W. A., Haub J. & Carter A. A cladding-pumped, tunable holmium doped fiber laser. Opt Express 21, 28415–28422 (2013). 24514352 10.1364/OE.21.028415

[b28] Peng W. J. . 1.94 μm Switchable Dual-Wavelength Tm 3+ Fiber Laser Employing High-Birefringence Fiber Bragg Grating. Appl Optics 52, 4601–4607 (2013). 10.1364/AO.52.004601 23842257

[b29] Zhang H., Xiao H., Zhou P., Wang X. & Xu X. High Power Yb-Raman Combined Nonlinear Fiber Amplifier. Opt Express 22, 10248–10255 (2014). 24921728 10.1364/OE.22.010248

[b30] Zhang L., Jiang H., Cui S. & Feng Y. Integrated ytterbium-Raman Fiber Amplifier. Opt Lett 39, 1933–1936 (2014). 24686642 10.1364/OL.39.001933

